# Dihydromyricetin Nanoparticles Alleviate Lipopolysaccharide-Induced Acute Kidney Injury by Decreasing Inflammation and Cell Apoptosis via the TLR4/NF-κB Pathway

**DOI:** 10.3390/jfb15090249

**Published:** 2024-08-29

**Authors:** Hongmei Yin, Qiaohua Yan, Yinglun Li, Huaqiao Tang

**Affiliations:** 1 School of Animal Science, Xichang University, Xichang 615012, China; 2 School of Veterinary Medicine, Sichuan Agricultural University, Chengdu 611130, China

**Keywords:** acute kidney injury, inflammation, sepsis, apoptosis

## Abstract

Acute kidney injury (AKI) is the most severe and fatal complication of sepsis resulting from infectious trauma. Currently, effective treatment options are still lacking. Dihydromyricetin is the main component extracted from Vine tea (*Ampelopsis megalophylla Diels et Gilg*). In our previous research, chitosan–tripolyphosphate-encapsulated nanoparticles of dihydromyricetin (CS-DMY-NPs) have been proven to have potential protective effects against cisplatin-induced AKI. Here, we investigated the protective effects and mechanisms of DMY and its nano-formulations against LPS-induced AKI by assessing pathological and inflammatory changes in mice. In mice with LPS-AKI treated with 300 mg/kg CS-DMY-NPs, the levels of creatinine (Cr), blood urea nitrogen (BUN), and KIM-1 were significantly reduced by 56%, 49%, and 88%, respectively. CS-DMY-NPs can upregulate the levels of GSH, SOD, and CAT by 47%, 7%, and 14%, respectively, to inhibit LPS-induced oxidative stress. Moreover, CS-DMY-NPs decreased the levels of IL-6, IL-1β, and MCP-1 by 31%, 49%, and 35%, respectively, to alleviate the inflammatory response. TUNEL and immunohistochemistry showed that CS-DMY-NPs reduced the number of apoptotic cells, increased the Bcl-2/Bax ratio by 30%, and attenuated renal cell apoptosis. Western blot analysis of renal tissue indicated that CS-DMY-NPs inhibited TLR4 expression and downregulated the phosphorylation of NF-κB p65 and IκBα. In summary, DMY prevented LPS-induced AKI by increasing antioxidant capacity, reducing inflammatory responses, and blocking apoptosis, and DMY nanoparticles were shown to have a better protective effect for future applications.

## 1. Introduction

AKI is a heterogeneous condition characterized by a sudden deterioration in renal function. It manifests as a sudden decrease in the glomerular filtration rate, elevated Cr concentration, or oliguria [1,2]. Multiple factors lead to AKI, including ischemia, sepsis, the use of diagnostic iodinated contrast agents, and the administration of aminoglycoside antibiotics [3]. Globally, there are estimated to be approximately 13 million cases annually, resulting in approximately 1.7 million deaths [4]. AKI has become a global public health issue, with high mortality rates and significant socioeconomic burdens due to the lack of effective treatments.

Sepsis, which is associated with a systemic inflammatory response, is caused by the invasion of pathogenic microorganisms such as bacteria. Sepsis often leads to multiorgan damage, and the kidney is the most commonly affected organ [5]. Lipopolysaccharides (LPSs) are a crucial component of Gram-negative bacteria and can be used to induce septic AKI. Studies have shown that TLR4 is the receptor for LPS and plays a critical role in the pathogenesis of LPS-induced septic AKI [6]. Moreover, NF-κB is a downstream transcription factor of the TLR4 signaling pathway. Activation of NF-κB increases the production of pro-inflammatory cytokines such as TNF-α, IL-1β, and IL-6 [7]. Excessive inflammatory responses and oxidative stress further induce cell apoptosis, leading to severe AKI [8]. Recent studies have shown that inflammatory cytokines such as IL-6 and TNF-α are associated with an increased risk of mortality in AKI patients; alleviating excessive inflammatory states is effective in treating AKI [9].

DMY, a natural dihydro-flavonol compound, is isolated from the leaves and tender stems of *Ampelopsis grossedentata* (Vine tea) [10]. Modern research has revealed various pharmacological properties of DMY, including antimicrobial, anti-inflammatory, anticancer, antioxidant, and antidiabetic activities [11]. DMY exhibits limited solubility, being soluble only in ethanol and hot water. The bioavailability of DMY is only about 4.02% in rats and may be <10% in humans [12]. These are the decisive factors that limit the pharmacological action and clinical application of DMY. Nanodrug carriers based on chitosan–tripolyphosphate have shown good delivery performance for poorly soluble drugs [13]. In our previous study, we prepared chitosan–tripolyphosphate-encapsulated nanoparticles of DMY to improve its stability, solubility, and bioavailability and demonstrated their protective effects in a mouse model of cisplatin-induced AKI [14]. Additionally, DMY exhibited protective effects on LPS-treated HK2 cells, reducing apoptosis and oxidative stress damage in these cells [15]. DMY has potential renal protective effects to reduce inflammation and oxidative stress, but its ability to prevent LPS-induced AKI in mice remains unknown. Therefore, this study aims to investigate the protective effects and mechanisms of DMY and its nano-formulation against septic AKI.

## 2. Materials and Methods

### 2.1. Chemicals and Antibodies

Dihydromyricetin (greater than 98%) was obtained from Shanghai Yuanye Bio-Technology Co., Ltd. (Shanghai, China). CS-DMY-NPs were prepared according to methods previously published by our team [14]. LPS was purchased from Solarbio Co., Ltd. (Beijing, China). Primary antibodies against NF-κB p65, IκBα, Caspase-3, Bax, Bcl-2, TLR4, and β-actin were purchased from Servicebio (Wuhan, China). Primary antibodies against NF-κB p-p65 and p-IκBα were obtained from Proteintech Group (Wuhan, China).

### 2.2. Animal Experiments

All animal experiments conformed to the guidance of the Animal Welfare and Ethics Committee of Sichuan Agricultural University (Approval No. 20230045). ICR mice (18–20 g, 6 weeks old), both male and female, were purchased from SPF Biotechnology Co., Ltd. (Beijing, China) and were housed in a 12 h light–dark cycle environment at room temperature 23 ± 1 °C. These animals had free access to food and water. Six groups were set (*n* = 8, with an equal number of male and female mice per group) as the control (saline) model (LPS only, 10 mg/kg), DMY water suspension (300 mg/kg), and LPS (10 mg/kg) + CS-DMY-NP (300, 200, and 100 mg/kg) groups. After 5 days of DMY treatment, the mice were intraperitoneally injected with LPS (10 mg/kg) to induce AKI; 24 h later, the mice were euthanized. Serum and kidney samples were collected for further analysis.

### 2.3. Assessment of BUN and Cr

The Cr and BUN test kits (Nanjing Jiancheng Bioengineering Institute, Nanjing, China) were used to determine the serum biomarker of renal function. The detection operation was in accordance with the manufacturer’s instructions.

### 2.4. Measurements of Antioxidant Enzyme Activity

The levels of antioxidative enzymes (CAT, SOD and GSH) were measured using commercially available kits (Nanjing Jiancheng Bioengineering Institute, Nanjing, China). To measure the activity of these enzymes, mouse kidney homogenate was tested following the instructions supplied by the manufacturer.

### 2.5. Measurement of Inflammatory Cytokines

Serum inflammatory cytokines (TNF-α, IL-1β, and IL-6) were tested by using commercial ELISA kits (Ruixin Industrial Co., Ltd., Quanzhou, China). The detection operation was in accordance with the manufacturer’s instructions.

### 2.6. Renal Histological Studies

Mouse kidney tissues were harvested, fixed and embedded in paraffin. Thin sections (μm) were cut and stained (H&E). The visualized morphological changes in the cellular and tissue structure were observed and photographed using a light microscope.

### 2.7. qRT-PCR

TransZol Up (TransGen Biotech, Beijing, China) was used to extract the Total RNA from the kidney, and cDNA was synthesized by using a reverse transcription kit (Applied Biological Materials, Vancouver, BC, Canada). qRT-PCR was conducted on a LightCycler^®^ 480II Master Mix (Roche, Munich, Germany) machine with a 10 μL reaction volume. The primer pairs are recorded in Table 1.

### 2.8. TUNEL Staining

Sections of 4 μm thick embedded kidney tissue were prepared and subjected to deparaffinization and rehydration. Proteinase K was used for antigen retrieval. Following antigen retrieval, a membrane-breaking solution was applied for permeabilization. Subsequently, the buffer was added and incubated at room temperature for 10 min. A mixture of the TDT enzyme, dUTP, and buffer at a ratio of 1:5:50 was prepared and applied to immerse the tissue. The cells were counterstained with DAPI for nuclear visualization, and the slides were mounted with an anti-fade mounting medium. Under a microscope, blue fluorescence of DAPI-stained nuclei was observed, while the nuclei of target-positive apoptotic cells exhibited green fluorescence.

### 2.9. Immunohistochemistry

According to the experimental protocol, antigen retrieval was performed using citrate buffer (pH 6.0). After antigen retrieval, the sections were placed in a 3% hydrogen peroxide solution. The sections were then blocked with 3% BSA, and the blocking solution was removed by flicking. Primary antibodies were incubated at 4 °C overnight, followed by the addition of HRP-conjugated secondary antibodies, and were incubated at room temperature for 50 min. The sections were washed with PBS (pH 7.4) on a decolorization shaker. DAB chromogen solution was applied and the sections were monitored under a microscope until brownish-yellow positive cells appeared, at which point tap water was used to stop color development. Hematoxylin was used to counterstain the nuclei, and a bluing reagent was applied, followed by rinsing under running water. Finally, the sections were dehydrated with ethanol and mounted. Under the microscope, the target cellular proteins appeared brownish-yellow, while the hematoxylin-stained nuclei were blue.

### 2.10. Western Blot Analysis

The kidney tissue was washed with precooled PBS and cut into small pieces. The tissue was then placed in a tissue grinder with grinding beads for homogenization. To ensure complete tissue lysis, the homogenate was kept on ice for an additional 30 min. The homogenate was subsequently centrifuged at 12,000 r/min for 10 min at 4 °C, after which the supernatant was collected. The protein concentration was tested using a BCA protein assay kit. The proteins were denatured and stored at −20 °C for later use. According to the calculated loading amount, proteins were loaded into wells for electrophoresis. After electrophoresis, the proteins were transferred onto a membrane. The 0.45 μm PVDF membrane was activated with methanol for 2 min before use. The transfer was carried out at a constant current of 300 mA for 30 min. The transferred membrane was quickly washed in TBST and then mixed with 5% milk at room temperature. The PVDF membrane was incubated with the primary antibody dilution on a shaker at 4 °C overnight. After the secondary antibodies were diluted in TBST (caspase-3, 1:3000; Bax, 1:500; Bcl-2, 1:1000; p-65, 1:3000; p-p65, 1:3000; TLR4, 1:3000; IκBa, 1:3000; p-IκBa, 1:3000), the membrane was incubated at room temperature for 30 min. The membrane was quickly rinsed with TBST, followed by a quick wash on a decolorization shaker. After washing, the PVDF membrane was dried and placed on a chemiluminescence imager tray. The mixed ECL detection reagent was added; after the reaction, the membrane was placed in a chemiluminescence imager (Thermo Fisher Scientific, Waltham, MA, USA) to visualize the target bands. Protein densitometry analysis was performed using ImageJ version 2.1.

### 2.11. Statistical Analysis

All the data are presented as the means ± SDs and analyzed by one-way ANOVA with Tukey’s post hoc test (IBM SPSS statistics 25, IBM, Armonk, NY, USA). The following marker was used to denote statistical significance: * *p* < 0.05; ** *p* < 0.01.

## 3. Results

### 3.1. DMY Alleviates LPS-Induced AKI in Mice

**Figure 1:**
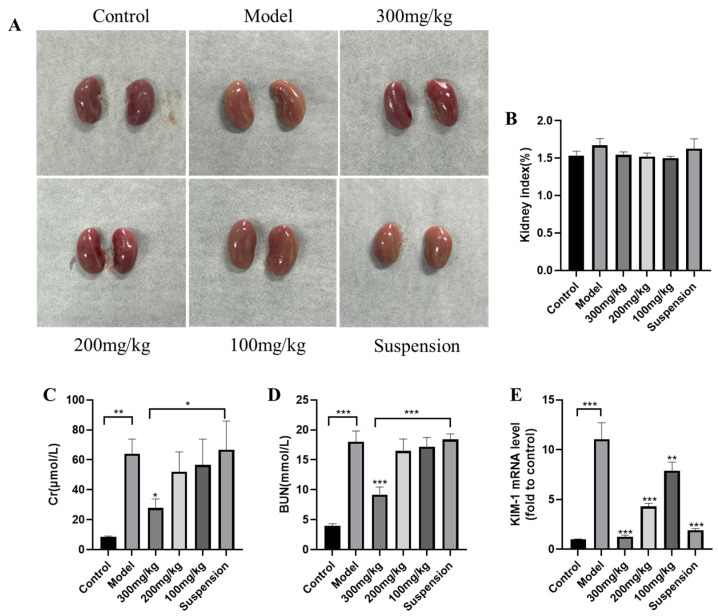
CS-DMY-NPs alleviated LPS-induced AKI. Kidney visual lesion (**A**), kidney index (**B**), Cr (**C**), and BUN (**D**) levels and mRNA expression levels of KIM-1 (**E**). All the data are presented as the means ± SD; * *p* < 0.05, ** *p* < 0.01, *** *p* < 0.001.

In addition to Cr and BUN, KIM-1 is a new and more sensitive biomarker for kidney function [16]. Normal kidneys appear reddish-brown. After LPS treatment, the kidneys appear pale and slightly larger, but the kidney index is not significant (Figure 1A,B). We measured the levels of Cr and BUN in the serum to evaluate the preliminary therapeutic effect of CS-DMY-NPs on LPS-induced AKI in mice. Where LPS caused AKI, it led to significant increases in Cr and BUN. Administration of high doses of CS-DMY-NPs resulted in a significant reduction in Cr and BUN levels, whereas the Cr and BUN levels did not significantly change in the suspension group (Figure 1C,D). Additionally, qRT-PCR was performed to detect the mRNA expression levels of KIM-1 (Figure 1E). Following the intraperitoneal injection of LPS, the expression of KIM-1 increased rapidly. The CS-DMY-NPs and DMY suspensions had similar effects on reducing KIM-1.

### 3.2. Antioxidant Enzyme Detection

**Figure 2:**
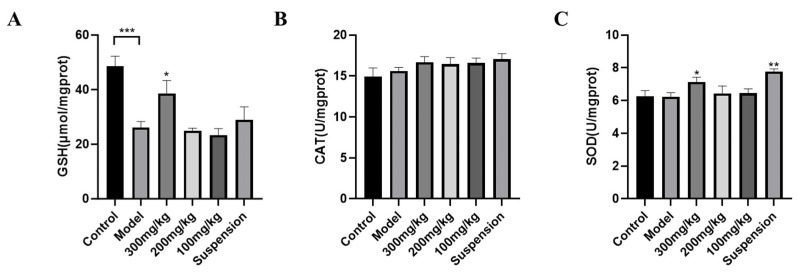
CS-DMY-NPs alleviated the oxidative stress induced by LPS. GSH (**A**), CAT (**B**) and SOD (**C**) levels in the kidneys. All the data are presented as the means ± SD; * *p* < 0.05, ** *p* < 0.01, *** *p* < 0.001.

As shown in Figure 2, LPS significantly decreased the release of GSH levels but had no significant influence on the SOD or CAT levels. Compared to the control treatment, the high dose of CS-DMY-NPs significantly alleviated the decreases in the GSH levels, and the GSH levels significantly increased in the serum of the mice. SOD activity significantly increased in the suspension group. Additionally, a high dose of CS-DMY-NPs had a greater effect on GSH accumulation in AKI mice than did the suspension treatment. However, LPS and CS-DMY-NPs had no significant effect on CAT activity.

### 3.3. Detection of Inflammatory Cytokines in Serum and Tissue

**Figure 3:**
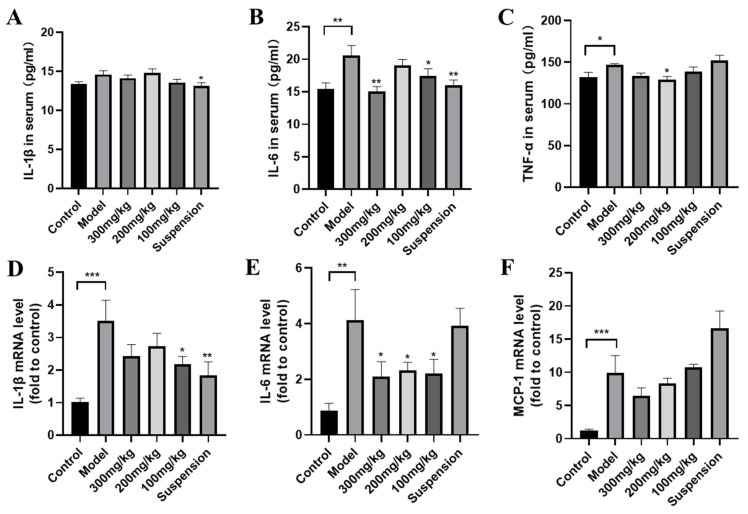
CS-DMY-NPs blocked the release of inflammation factors induced by LPS. IL-6 (**A**), IL-1β (**B**), and TNF-α (**C**) levels in serum and mRNA expression levels of IL-1β (**D**), IL-6 (**E**), and MCP-1 (**F**) in kidney tissue. All the data are presented as the means ± SD; * *p* < 0.05, ** *p* < 0.01, *** *p* < 0.001.

As shown in Figure 3, after intraperitoneal injection of LPS, the levels of IL-6 and TNF-α in the serum significantly increased, while the levels of IL-1β, although not significantly different, still increased, and DMY decreased the levels of inflammatory factors in the serum. The qRT-PCR results demonstrated a similar increase in inflammatory factor levels in mouse kidney tissues. MCP-1 significantly increased under LPS stimulation, and although not significantly, high-dose CS-DMY-NP treatment still reduced MCP-1. These results suggest that DMY effectively alleviates renal inflammation induced by LPS.

### 3.4. Histopathological Analysis

**Figure 4:**
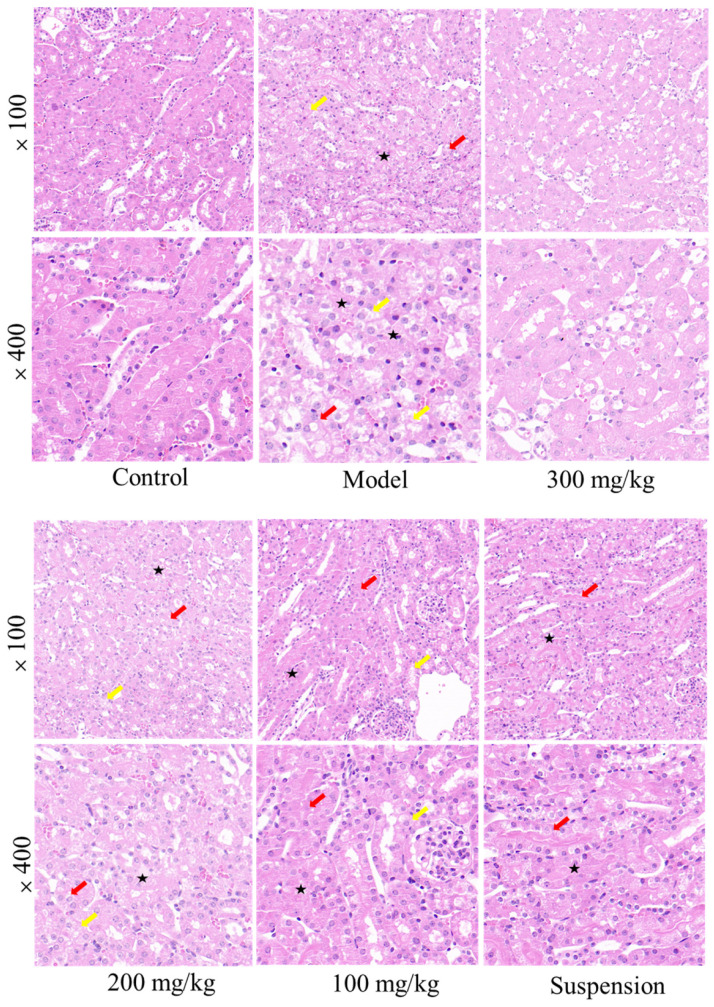
H&E staining of mouse kidney tissue. The red arrow indicates stenosis of the renal tubule. The black five-pointed star represents the swelling of renal tubular epithelial cells; the yellow arrows represent tubular epithelial vacuoles.

LPS administration results in pathological damage to the kidneys, including tubular dilation, tubular epithelial vacuolation, loss of brush border, and exposure of renal epithelial nuclei [17]. As shown in Figure 4, the model group exhibited tubular swelling, luminal narrowing, and tubular vacuolation in the kidneys. Following treatment with 300 mg/kg CS-DMY-NPs, no significant renal lesions or tubular vacuolation were observed. However, the same dose of DMY suspension still resulted in some degree of tubular swelling and vacuolar degeneration of the renal epithelium.

### 3.5. DMY Decreased the TUNEL Staining Signal

**Figure 5:**
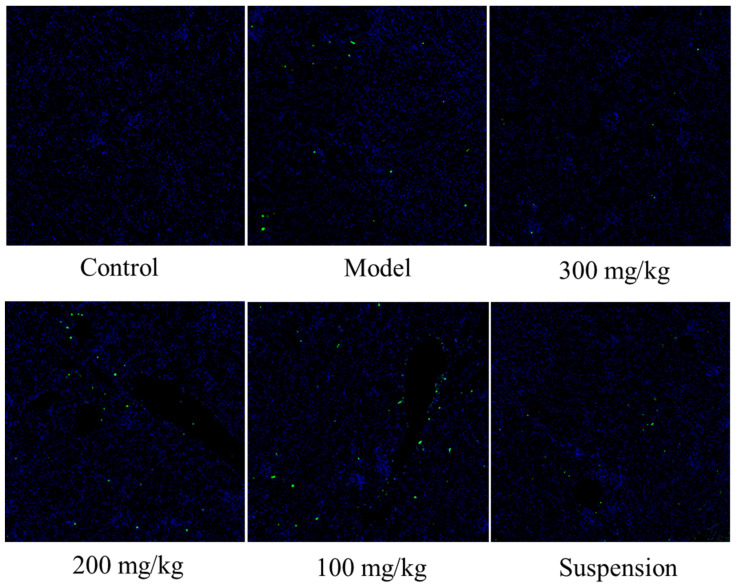
Detection of kidney cell apoptosis by TUNEL staining (×25 magnification).

To investigate the influence of CS-DMY-NPs on renal cell apoptosis, TUNEL staining was used to evaluate the cell apoptosis by observing the intensity of green fluorescence. As shown in Figure 5, LPS induced cell apoptosis, whereas CS-DMY-NPs significantly reduced the number of TUNEL-positive cells in AKI mice. The TUNEL results demonstrated that CS-DMY-NPs reduced the number of apoptotic cells in the kidney.

### 3.6. CS-DMY-NPs Reduced Cell Apoptosis in the Kidneys

**Figure 6:**
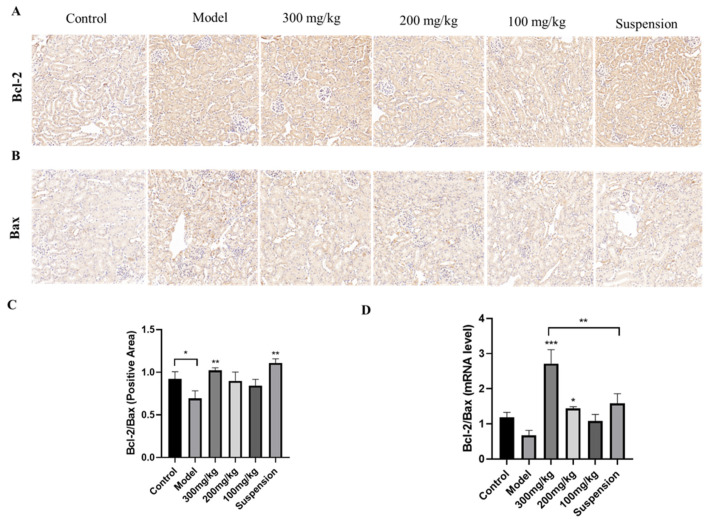
The regulatory effect of CS-DMY-NPs on the Bcl-2 and Bax pathways. Immunohistochemical analysis of Bcl-2 (**A**) and Bax (**B**), immunohistochemical quantification of Bcl-2 and Bax (**C**), and mRNA expression ratios of Bcl-2 and Bax (**D**). All the data are presented as the means ± SD; * *p* < 0.05, ** *p* < 0.01, *** *p* < 0.001.

The expression levels of Bcl-2 and Bax in renal tissue were assessed via immunohistochemistry (Figure 6A,B). In the control group, both Bcl-2 and Bax exhibited low expression levels. Following LPS injection, Bax protein expression significantly increased, whereas Bcl-2 expression was only slightly elevated. After treatment, Bax expression markedly decreased in a dose-dependent manner, with minimal impact on Bcl-2 levels. The Bcl-2/Bax ratio was calculated; it showed a significant increase in the model group and a dose-dependent decrease in the treatment group (Figure 6C). The qRT-PCR results further indicated that CS-DMY-NPs reduced Bax mRNA expression(Figure 6D), consistent with the immunohistochemistry findings. The results suggested that DMY has a more pronounced effect on Bax, and administering high doses of CS-DMY-NPs can significantly reduce Bax expression, thereby alleviating LPS-induced apoptosis in mouse renal cells.

### 3.7. The Impact of CS-DMY-NPs on Apoptotic Signaling Pathways

**Figure 7:**
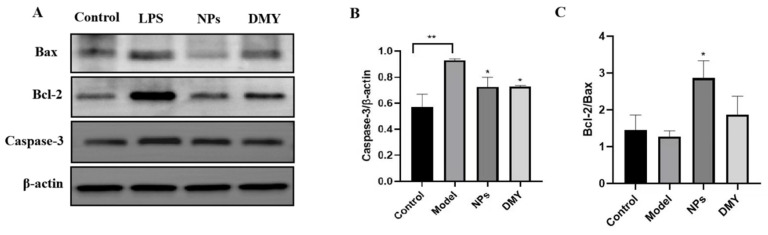
The impact of CS-DMY-NPs on apoptotic signaling pathways. Protein bands of Bax, Bcl-2, and Caspase-3 (**A**), grayscale intensity chart of Caspase-3 (**B**), and grayscale intensity ratios of Bcl-2 and Bax (**C**). All the data are presented as the means ± SD; * *p* < 0.05, ** *p* < 0.01.

Bcl-2 family proteins and caspase family proteins are key regulators of cell apoptosis. Caspase-3 is a central kinase in the caspase family that, upon activation, can initiate a caspase cascade leading to cell apoptosis [18]. To determine the effect of CS-DMY-NPs on apoptotic proteins, the expression of Bax, Bcl-2, and caspase-3 was analyzed using Western blotting. As shown in Figure 7, CS-DMY-NPs significantly reduced the LPS-induced overexpression of caspase-3. The Bcl-2/Bax ratio determines a cell’s susceptibility to apoptosis, with a higher ratio indicating stronger antiapoptotic effects [19]. The model group showed little change compared to the control group, likely due to the body’s inherent repair mechanisms. However, after treatment with CS-DMY-NPs, the ratio increased significantly.

### 3.8. The Impact of CS-DMY-NPs on the TLR4/NF-κB Signaling Pathway

**Figure 8:**
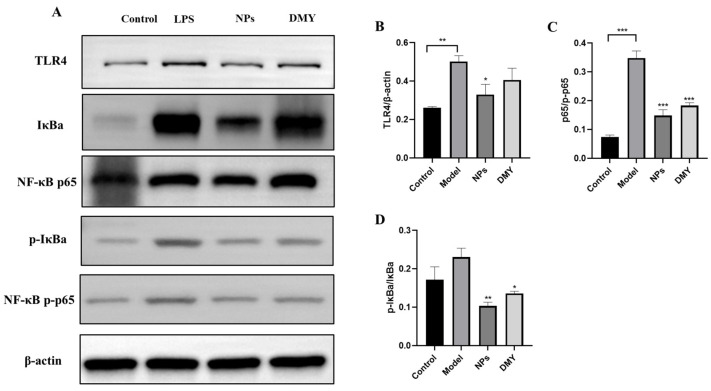
The impact of CS-DMY-NPs on the TLR4/NF-κB signaling pathway. Protein bands of TLR4, IκBa, and NF-κB (**A**) and grayscale intensity charts of TLR4, p-IκBa/IκBa, and NF-κB p-p65/p65 (**B**–**D**). All the data are presented as the means ± SD; * *p* < 0.05, ** *p* < 0.01, *** *p* < 0.001.

To elucidate the anti-inflammatory mechanism of CS-DMY-NPs in LPS-induced septic AKI mouse kidneys, further investigation into the activation of the signaling pathway was conducted through Western blot analysis. As shown in Figure 8, LPS stimulation significantly activated TLR4, NF-κB, and IκBα phosphorylation, leading to the degradation of IκBα. These results suggest that CS-DMY-NPs have the potential to inhibit the activation of the TLR4/NF-κB pathway in AKI mice induced by LPS.

## 4. Discussion

Bacterial virulence is one of the most dangerous factors contributing to the development of septic AKI. LPSs, the main pathogenic factor in Gram-negative bacterial infections, trigger a robust inflammatory response in the body [20]. Septic AKI is characterized by complex pathogenic mechanisms, including infiltration of inflammatory mediators, dysfunction of renal vascular endothelial cells, and apoptosis of renal cells [21]. Currently, there are no specific drugs for treating sepsis-induced AKI. This study indicates that DMY can alleviate AKI induced by LPS in mice, and the effect greatly improves when it is prepared in the form of nanoparticles.

This study has demonstrated that high-dose CS-DMY-NPs significantly regulated the release of IL-1β, TNF-α, and IL-6 in the kidneys of mice, suggesting that DMY may attenuate LPS-induced acute kidney inflammation injury. Furthermore, histological analysis confirmed that DMY alleviated LPS-induced kidney damage. These results suggest that DMY also has a protective effect against LPS-induced AKI. The inflammation induced by LPS can trigger the release of numerous pro-inflammatory cytokines, which are considered to be a direct trigger of renal tubular epithelial cell injury, one of the most important pathogenic mechanisms of AKI [22]. TNF-α is a key mediator of sepsis that can induce kidney injury by activating TNF receptors. IL-1β plays a major role in local acute inflammation, while IL-6 is considered to be a predictor of AKI in critically ill septic patients [23]. These inflammatory cytokines initiate and amplify the inflammatory response, leading to the occurrence of AKI [24]. In previous mechanism research studies, DMY was shown to inhibit the activation of NOD-like receptor protein 3, nuclear factor kappa-B, and mitogen-activated protein kinase signaling pathways to block the intense release of inflammatory products induced by LPS [25,26]. The nano-formulated DMY would provide a higher kidney drug concentration, which showed that CS-DMY-NPs have a better anti-inflammatory effect against AKI.

TLR4 serves as a pattern recognition receptor and functions as a sensor for LPS. Activation of TLR4 leads to the recruitment of inflammatory factors and subsequent renal damage [27]. Moreover, the binding of TLR4 to LPS induces the activation of NF-κB, a crucial transcription factor that plays a pivotal role in inflammation. Activated NF-κB translocates to the nucleus and stimulates the release of inflammatory mediators, such as IL-1β, IL-6, and TNF-α [28]. Under inflammatory stimulation, the IκBa protein is phosphorylated, ubiquitinated, and degraded. Degradation of IκBa allows NF-κB proteins to regulate the transcription of inflammatory regulation genes in the nucleus [29]. Inhibiting the TLR4/NF-κB-mediated inflammatory response has been shown to have a renoprotective effect against AKI [30]. Our data indicate that DMY can suppress LPS-induced NF-κB activation by downregulating the expression of p-p65 and p-IκBα. In addition, the effect of CS-DMY-NPs is more significant. These results are consistent with previous research results that DMY significantly inhibits NF-κB (IκBα) phosphorylation and degradation, as well as subsequent nuclear translocation of p65 [31].

The results indicate that LPSs deplete antioxidant enzymes in mice, whereas CS-DMY-NPs mitigate the depletion of GSH and SOD, significantly increasing their levels in mouse serum. Oxidative stress can induce the expression of multiple pro-inflammatory cytokines. The body’s antioxidant defense system can clear excessive released free radicals efficiently and inhibit the production of lipid peroxidation to protect cells [32]. Studies have reported an increase in lipid oxidation biomarkers and a decrease in antioxidant enzyme activity in septic patients [33]. DMY can activate ERK/Nrf2/HO-1 signaling pathway to upregulate the antioxidant capacity in cells and organisms [34,35].

The results of this study demonstrate that CS-DMY-NPs reduce the number of apoptotic cells and increase the ratio of Bcl-2 to Bax proteins. These results confirm the significant inhibitory effect of CS-DMY-NPs on LPS-induced cell apoptosis. Although there is some controversy regarding the role of tubular cell apoptosis in organ damage associated with sepsis, cell apoptosis has been recognized as an important pathogenic mechanism of septic AKI [36]. Bcl-2 and Bax can interact to regulate the release of cytochrome C and further activate the cell apoptosis [37]. There are many studies that have shown a clear relationship between oxidative stress, inflammation and apoptosis, which indicates that DMY may cause different results due to the activation process [38,39]. Here, DMY can reduce LPS-induced cell apoptosis mainly by inhibiting the oxidative stress and inflammation in AKI.

In summary, DMY has a protective effect on LPS-induced AKI in mice. After it is prepared in the form of nanoparticles, its anti-inflammatory activity, antioxidant capacity, and anti-apoptotic ability are all improved. This means that DMY is a potential drug for the treatment of AKI, and the nanoparticles we prepared provide a reference for the clinical use of DMY.

## 5. Conclusions

In conclusion, CS-DMY-NPs exhibited the potential to inhibit oxidative stress and pro-inflammatory cytokines, significantly reducing renal cell apoptosis, and thereby improving LPS-induced renal dysfunction and histological damage. The protective mechanism involves the inhibition of renal cell apoptosis and the downregulation of p-p65 and p-IκBα expression via the TLR4/NF-κB pathway. Our study indicates that CS-DMY-NPs exhibited protective effects on septic AKI and that the effect is greater than that of the DMY suspension.

## Figures and Tables

**Table 1:** Primers used in qRT-PCR.

Genes	Type	Sequences (5→3)
*KIM-1*	Fw	CTGGAATGGCACTGTGACATCC
	Rev	GCAGATGCCAACATAGAAGCCC
*IL-1β*	Fw	GCAACTGTTCCTGAACTCAACT
	Rev	ATCTTTTGGGGTCCGTCAACT
*MCP-1*	Fw	CATCCACGTGTTGGCTCA
	Rev	GATCATCTTGCTGGTGAATGAGT
*IL-6*	Fw	AAAGAGTTGTGCAATGGCAATTCT
	Rev	AAGTGCATCATCGTTGTTCATACA
*Bcl-2*	Fw	TGTGAGGACCCAATCTGGAAA
	Rev	TTGCAATGAATCGGGAGTTG
*Bax*	Fw	GATCAGCTCGGGCACTTTAG
	Rev	TTGCTGATGGCAACTTCAAC
*NF-κB*	Fw	ATGTGGAGATCATTGAGCAGC
	Rev	CCTGGTCCTGTGTAGCCATT
*GAPDH*	Fw	AGGTCGGTGTGAACGGATTTG
	Rev	TGTAGACCATGTAGTTGAGGTCA

## Data Availability

The original contributions presented in the study are included in the article, further inquiries can be directed to the corresponding author.
